# Probabilistic mapping and automated segmentation of human brainstem white matter bundles

**DOI:** 10.1073/pnas.2509321123

**Published:** 2026-02-06

**Authors:** Mark D. Olchanyi, David R. Schreier, Jian Li, Chiara Maffei, Annabel Sorby-Adams, Hannah C. Kinney, Brian C. Healy, Holly J. Freeman, Jared Shless, Christophe Destrieux, Henry Tregidgo, Juan Eugenio Iglesias, Emery N. Brown, Brian L. Edlow

**Affiliations:** ^a^Neuroscience Statistics Research Laboratory, Massachusetts Institute of Technology, Cambridge, MA 02139; ^b^Center for Neurotechnology and Neurorecovery, Department of Neurology, Massachusetts General Hospital, Boston, MA 02114; ^c^Institute for Medical Engineering and Science, Massachusetts Institute of Technology, Cambridge, MA 02142; ^d^Department of Anesthesia, Critical Care and Pain Medicine, Massachusetts General Hospital and Harvard Medical School, Boston, MA 02114; ^e^Athinoula A. Martinos Center for Biomedical Imaging, Department of Radiology, Massachusetts General Hospital and Harvard Medical School, Charlestown, MA 02129; ^f^Department of Neurology, Massachusetts General Hospital and Harvard Medical School, Boston, MA 02114; ^g^Department of Pathology, Boston Children’s Hospital and Harvard Medical School, Boston, MA 02115; ^h^Department of Biostatistics, T.H Chan School of Public Health, Harvard University, Boston, MA 02115; ^i^Imaging Brain and Neuropsychiatry iBraiN U1253, Université de Tours, INSERM, Tours 37032, France; ^j^Centre Hospitalier Régional Universitaire de Tours, Tours 37044, France; ^k^Hawkes Institute, University College London, London WC1V 6LJ, United Kingdom; ^l^Computer Science and AI Laboratory, Massachusetts Institute of Technology, Cambridge, MA 02139; ^m^The Picower Institute for Learning and Memory, Department of Brain and Cognitive Science, Massachusetts Institute of Technology, Cambridge, MA 02139

**Keywords:** diffusion MRI, tractography, brainstem, machine learning, segmentation

## Abstract

Brainstem white matter (WM) bundles are essential conduits for neural signals that modulate homeostasis and consciousness. Their architecture forms the anatomic basis for brainstem connectomics, subcortical circuit models, and deep brain navigation tools. However, their small size and complex morphology, compared to cerebral WM, makes mapping and segmentation challenging in neuroimaging. As a result, fundamental questions about brainstem modulation of human homeostasis and consciousness remain unanswered. We leverage diffusion MRI tractography to create BrainStem Bundle Tool (BSBT), which automatically segments eight WM bundles in the rostral brainstem. BSBT performs segmentation on a custom probabilistic fiber map using a convolutional neural network architecture tailored to detect small anatomic structures. We demonstrate BSBT’s robustness across diffusion MRI acquisition protocols with in vivo scans of healthy subjects and ex vivo scans of human brain specimens with corresponding histology. BSBT also detected distinct brainstem bundle alterations in patients with Alzheimer’s disease, Parkinson’s disease, multiple sclerosis, and traumatic brain injury through tract-based analysis and classification tasks. Finally, we provide proof-of-principle evidence for the prognostic utility of BSBT in a longitudinal analysis of traumatic coma recovery. BSBT creates opportunities for scalable mapping of brainstem WM bundles and investigation of their role in a broad spectrum of neurological disorders.

## Significance

Vital brainstem functions are relayed through clustered myelin-coated axons termed white matter (WM) bundles. There is presently no reliable method for delineating these brainstem structures, largely due to their morphological complexity. We map WM contrast and create a neural network model to automatically trace eight brainstem WM bundles in diffusion MRI. We validate this methodology with in vivo and ex vivo diffusion MRI data and demonstrate that microstructural and morphologic changes in distinct subsets of these bundles are associated with Alzheimer’s disease, Parkinson’s disease, multiple sclerosis, and traumatic brain injury. Our method establishes a foundation for fully automated brainstem connectivity mapping, which will enhance our understanding of brainstem contributions to multiple neurological disorders.

The brainstem is a compact structure that orchestrates vital functions like respiration, circadian rhythm, cardiovascular homeostasis, and consciousness (1–5). These functions are modulated by brainstem white matter (WM) bundles, whose disruption is increasingly recognized as contributing to a vast range of neurological disorders (6–12). Human brainstem connectomics is an increasing area of focus in fields such as network neuroscience, deep brain stimulation, and disorders of consciousness (13–18), where accurate delineation of brainstem networks and their WM connections is essential for mechanistic insights and translational progress. With recent advances in noninvasive imaging tools such as diffusion MRI (dMRI) tractography (19, 20), brainstem network mapping is now possible to perform in vivo and noninvasively (15, 21, 22). However, because of their small size and complex branching patterns, mapping individual brainstem bundles remains largely unexplored. To address this methodologic barrier, we aimed to develop a fully automated method to analyze the morphology and structural integrity of brainstem bundles in both healthy brains and those impacted by disease.

Most prior brainstem mapping studies relied upon propagating streamlines between manually labeled structures with dMRI tractography (15, 17, 23, 24), a time-consuming approach that requires neuroanatomic expertise. Beyond manual labeling, dMRI segmentation of WM can be performed through semisupervised and supervised methods. Semisupervised approaches include tractography-based aggregation of fibers according to connectivity rules with proximal regions of interest (ROIs) (25, 26), and clustering algorithms that encode WM features initialized by user-defined parameters (27, 28). Fully supervised approaches, which benefit from increased accuracy with sufficient training data, include atlas registration with WM templates, segmentation of tractograms constrained by gray matter ROIs, and deep-learning models based on whole-brain tractography (29–34). These algorithms mainly segment large brainstem bundles, such as the corticospinal tracts and superior cerebellar peduncles (SCPs) (30, 32, 34). Most brainstem bundles are significantly smaller (24, 35), possess lower contrast (36), and are plagued by cardiorespiratory noise and pulsatile flow of cerebrospinal fluid which create off-resonance artifacts during in vivo MRI acquisition (37, 38) and undermine segmentation. The absence of segmentation tools for smaller bundles thus precludes the automated study of in vivo brainstem connectivity in healthy individuals and patients with neurological diseases affecting the brainstem.

Here, we developed a fully automated, unsupervised brainstem bundle segmentation method, termed BrainStem Bundle Tool (BSBT). BSBT segments eight WM bundles in the pons and midbrain directly from dMRI, without manual intervention. Segmentation is performed on low-b (b = 0 s/mm^2^) and fractional anisotropy (FA) channels, coupled with a streamline map generated from automated probabilistic tractography in the brainstem. BSBT utilizes a convolutional neural network (CNN) architecture for segmentation. The CNN possesses two elements that optimize identification of small structures: an attention gate situated between the three highest-resolution encoder and decoder layers, and a semidense conditional random field (CRF) at its *SoftMax* output. We assess BSBT robustness through ablation testing in one ex vivo and two in vivo dMRI datasets, as well as through in vivo dMRI test–retest analysis.

It is important to distinguish this study from our previous work, where we segmented brainstem gray matter nuclei (39). Both studies utilize a common subset of ex vivo brain specimens and in vivo MRI scans of traumatic brain injury (TBI) patients but characterize different brainstem properties. In the current study, we now segment brainstem WM. Our previous work leveraged the ex vivo non-diffusion-weighted MRI data to construct a probabilistic atlas of arousal nuclei. The current work uses dMRI contrast from the ex vivo specimens to localize WM bundles and validate the BSBT CNN model. In addition, we demonstrate the potential application of brainstem WM segmentation to a broad spectrum of neurological diseases. Specifically, we test BSBT performance in identifying changes in the diffusion characteristics and volume of brainstem bundles in four patient cohorts: Alzheimer’s disease (AD), Parkinson’s disease (PD), multiple sclerosis (MS), and TBI. Finally, we expand on our prior brainstem gray matter analysis of TBI patients and provide proof-of-principle evidence that BSBT and brainstem WM assessment yields insights that can potentially improve prognostication through longitudinal WM mapping in a patient with acute traumatic coma. These analyses show that BSBT can be a powerful tool to study brainstem connectivity and discover imaging biomarkers in neurological disorders with brainstem pathology.

## Results

### WM Bundle Selection With Automated Tractography.

For segmentation, we located brainstem bundles that displayed probabilistic fiber map (PFM) contrast boundaries in ex vivo and in vivo dMRI. Details on PFM construction are provided in the *Materials and Methods* and *SI Appendix*, *Supplementary Text*. With the aid of corresponding Hematoxylin and Eosin/Luxol Fast-Blue (HE-LFB) histological sections in two ex vivo brain specimens [which we previously used for probabilistic atlas construction of brainstem gray matter nuclei with neurotransmitter-specific immunostains (39)], we identified the pontine (caudal, c) and mesencephalic (rostral, r) divisions of the medial lemniscus (MLc/MLr), SCP, brainstem division of the lateral forebrain bundle (LFB), mesencephalic homeostatic bundle (MHB), brachium of the inferior colliculus (Bic), medial longitudinal fasciculus (MLF), and central tegmental tract (CTG) for segmentation.

**Fig. 1.**
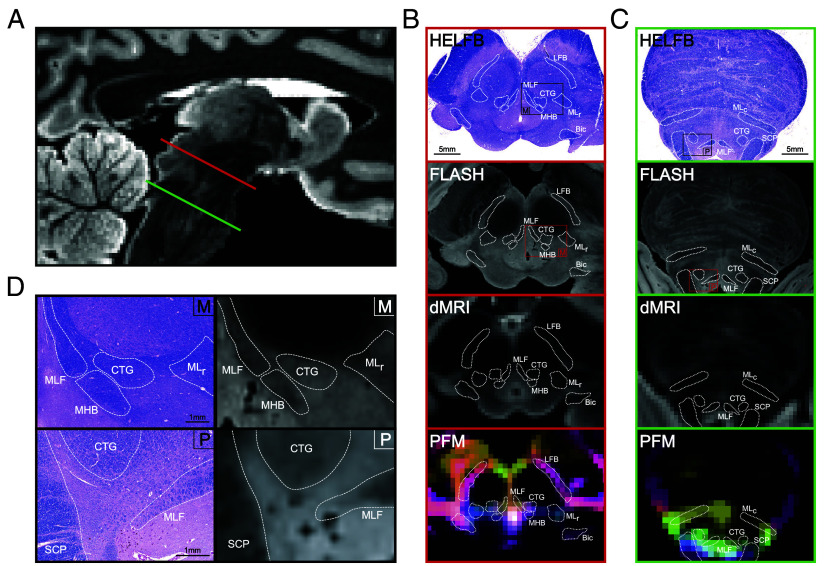
Histological mapping of white matter in the rostral brainstem and correlations with ex vivo MRI and Probabilistic Fiber Mapping. (*A*) A mid-sagittal view of a low-b map from an ex vivo brain specimen, with a representative HE-LFB-stained histological section cut along the axial plane of the midbrain as indicated by the red slice (*B*), and a representative HE-LFB pontine histological section as indicated by the green slice (*C*). For each section, corresponding brainstem bundle locations in the FLASH volume, low-b image, and PFM reconstruction are shown. (*D*) Zoomed midbrain (“M”) and pontine (“P”) histology views with corresponding FLASH contrasts. HE-LFB: Hematoxylin and eosin-luxol fast blue, PFM: Probabilistic fiber map MLc: Medial lemniscus (caudal), SCP: Superior cerebellar peduncle, LFB: Lateral forebrain bundle, MHB: Mesencephalic homeostatic bundle, Bic: Brachium of the inferior colliculus, MLr: Medial lemniscus (rostral), MLF: Medial longitudinal fasciculus, CTG: Central tegmental tract.

In ex vivo brains with histology, we observed PFM contrast corresponding with high-resolution Fast Low-Angle SHot (FLASH) MRI contrast and histological sections for each bundle (Fig. 1). In ex vivo brains without histology, we relied instead on FLASH contrast alone, for which histological correspondence is shown in Fig. 1*D*. We confirmed bundle identities with a human brainstem atlas (35). Further information on the neuroanatomic borders of brainstem bundles is in the *SI Appendix*, *Supplementary Text*. Due to the lack of LFB/MHB atlas regions, we confirmed their morphology with deterministic tractography, as illustrated in *SI Appendix*, Figs. S2 and S3. BSBT segmentations in a representative in vivo scan along with corresponding deterministic tracts are shown in *SI Appendix*, Fig. S4.

### Segmentation Accuracy Compared to Ground-Truth Manual Annotations.

To test BSBT accuracy across different resolutions and domains, we evaluated segmentations on ground-truth brainstem bundles manually annotated on in vivo dMRI of healthy subjects (n = 25) and ex vivo dMRI of normative brains (n = 7). The annotation process is detailed in the *SI Appendix*, *Supplementary Text*. In vivo dMRI scans were obtained from the Human Connectome Project (HCP) dataset (n = 15, 1.2 mm isotropic resolution, multishell dMRI) (40, 41) and from the control group of the Alzheimer’s Disease Neuroimaging Initiative 3 (ADNI3) dataset (n = 10, 2 mm isotropic resolution, single-shell dMRI) (42). Ex vivo brains (750 μm isotropic resolution, single-shell dMRI) were donated by patients without neurological disorders who died of nonneurological causes. Manual annotations and BSBT segmentations for all validation cases are released via our OpenNeuro repository, and illustrated in *SI Appendix*, Fig. S5. Clinical/demographic information for each ex vivo brain is provided in *SI Appendix*, Table S1. Acquisition details for all dMRI data are in the *SI Appendix*, *Supplementary Text*.

**Fig. 2.**
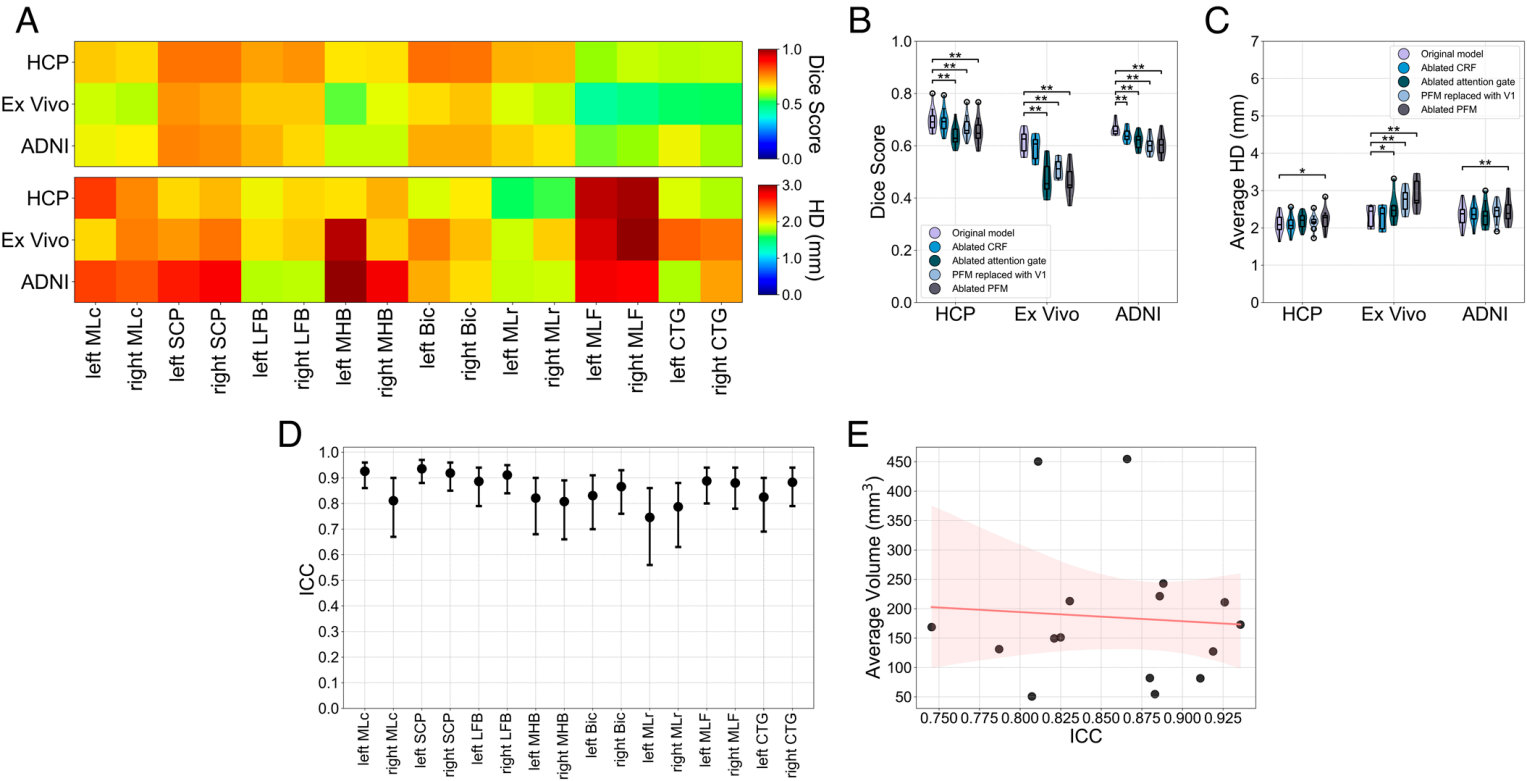
Accuracy, ablation, and test–retest reliability analysis for BSBT segmentations. (*A*) A heat map of Dice scores (*Top*) and HD (*Bottom*) for each brainstem bundle segmentation compared to manual annotations (averaged across subjects) in HCP subjects, ex vivo brain specimens, and ADNI3 control subjects. (*B* and *C*) Violin plots with overlaid box plots consisting of per-subject Dice and HD (averaged across bundles) under various ablations. Significance bars indicate uncorrected two-tailed Wald tests from a linear mixed effects model: *P < 0.05 and **P < 0.01. (*D*) ICC values with 95% CI from a two-way ANOVA mixed effects model for BSBT-segmented bundle volumes from test–retest HCP subjects. (*E*) ICCs for each brainstem bundle plotted against their respective volumes (averaged across all subjects), with a linear regression fit (red line) and shaded 95% CI for the regression line. HD: Hausdorff distance, ICC: Intraclass correlation coefficient, MLc: Medial lemniscus (caudal), SCP: Superior cerebellar peduncle, LFB: Lateral forebrain bundle, MHB: Mesencephalic homeostatic bundle, Bic: Brachium of the inferior colliculus, MLr: Medial lemniscus (rostral), MLF: Medial longitudinal fasciculus, CTG: Central tegmental tract.

We used Dice scores and average Hausdorff distances (HD) as accuracy metrics for benchmarking BSBT against ground-truth annotations (Fig. 2*A*). Each metric is further described in the *SI Appendix*, *Supplementary Text*. Larger bundles by volume (e.g., SCP, LFB, and Bic) displayed comparatively higher Dice across all datasets. HDs were significantly less variable than Dice, and less dependent on bundle size. This observation is partially attributable to HD being a boundary metric that is less susceptible to fluctuations in overlap, especially between small labels. However, the lower HD variability across both resolution and bundle size indicates high spatial precision across modalities. Subject-averaged accuracy was better in HCP subjects (Dice = 0.70, HD = 2.11) compared to ADNI3 subjects (Dice = 0.66, HD = 2.34) and ex vivo brain specimens (Dice = 0.62, HD = 2.32). Finally, we synthetically downsampled all datasets and found that Dice/HD showed relative stability in accuracy until resolutions of 2.5-3 mm (*SI Appendix*, Fig. S6). Volumetric distributions for all BSBT-segmented bundles across validation subjects, as well as whole-brain masks for reference, are included in *SI Appendix*, Table S2.

### Segmentation Accuracy Under Ablated Conditions.

We performed ablation testing to assess the utility of core BSBT components in segmenting brainstem bundles. We individually removed the CRF and attention-gating mechanism from the CNN model to test the influence of *SoftMax* correction and attention modulation to localize small, clustered WM regions. We removed/replaced the PFM to test the value of vector diffusion maps, such V1 and probabilistic tractography, for encoding WM information. Dice/HDs for each ablation are shown in Fig. 2 *B* and *C*.

The unablated CNN generally showed greater segmentation performance than did each ablated counterpart. Attention gate removal degraded accuracy across all datasets (average Dice reduction of 0.08 and 0.1 mm increase in HD), highlighting its importance in localizing variable-sized brainstem bundles. PFM removal resulted in the largest overall decrease in accuracy (average Dice reduction of 0.09 and 0.3 mm increase in HD). Compared to the unablated CNN, all uncorrected p-values for PFM ablation from a linear mixed-effects model were <0.01, except for HCP dataset HD (*P* = 0.04). Overall, drop-offs in accuracy were more significant in ex vivo data; uncorrected p-values for each ablation were <0.05, except for CRF removal (Dice *P* = 0.08, HD *P* = 0.61). These observations suggest that with poor diffusion contrast (i.e., fixed brain tissue/weak b-values), enhancing sensitivity to WM boundaries with attention gating and tractography may be critical to segmentation. Details on all statistical analysis are in the *SI Appendix*, *Supplementary Text*.

To assess how alternative feature encoding or training regimen affects segmentation, we trained additional CNN models where FA was replaced with other commonly derived diffusion scalars: mean diffusivity, axial diffusivity, and radial diffusivity. We also compared our Dice training loss to a boundary-aware “HD-like” loss: the L1 distance between the CNN pre-*SoftMax* logit layer and the ground-truth signed distance map. Using an identical 80%/20% train-validation split from the original HCP training data, all input variants converged to comparable accuracies, while both boundary-only training loss and Dice/boundary hybrid loss displayed worse accuracy (*SI Appendix*, Fig. S7).

### Test–Retest Reliability.

We analyzed a separate group of 40 HCP subjects who underwent two separate scanning sessions with the same protocol (average time between scans = 4.8±2.1 mo). These subjects served as a “test–retest” group, where longitudinal brainstem bundle volume changes were quantified with Intraclass correlation coefficients (ICC) to assess BSBT reliability. We observed high reliability for most brainstem bundles (ICC > 0.8, Fig. 2*D*). The left/right MLr displayed ICCs > 0.7 (ICC: left = 0.75, right = 0.79). ICCs did not correlate with bundle volumes ($R^{2}=0.005$, P=0.79) (Fig. 2*E*), suggesting that reliability remains consistent across bundle sizes.

### Evaluation of Diffusion and Volumetric Changes in Neurological Disorders.

**Fig. 3.**
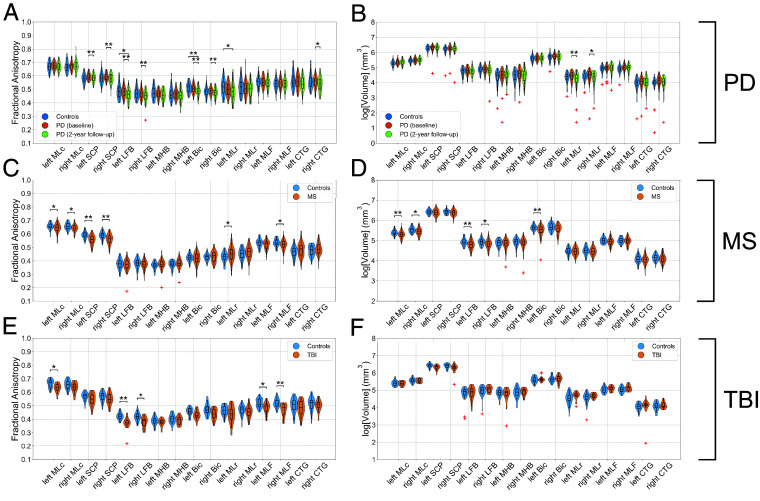
Violin plots of average fractional anisotropy (*Left*) and volume (*Right*) distributions for each brainstem bundle in control (blue), patient (orange), and two-year PD patient follow-up (green) groups for Parkinson’s disease (*A* and *B*), multiple sclerosis (*C* and *D*), and traumatic brain injury (*E* and *F*) cohorts. Significance bars indicate FDR-corrected two-tailed Wilcoxon rank-sum tests (or signed-rank tests for PD-2YFU comparisons): *P < 0.05 and **P < 0.01. PD: Parkinson’s disease, PD-2YFU: Parkinson‘s disease two-year follow-up, MS: Multiple sclerosis, TBI: Traumatic brain injury, FDR: False discovery rate.

**Fig. 4.**
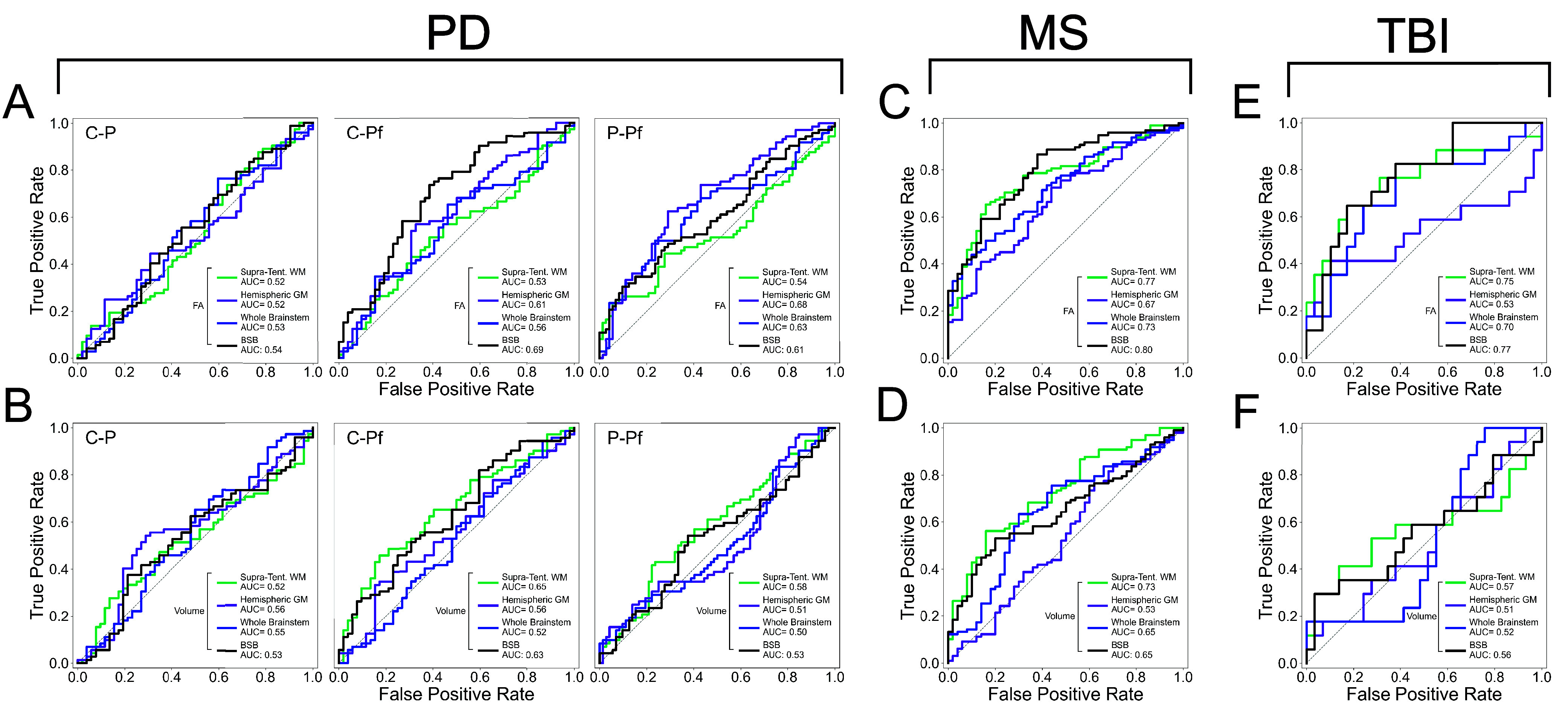
Receiver-operating characteristic curves for linear discriminant analysis classifiers trained on fractional anisotropy and volume to distinguish subjects in control versus patient groups in Parkinson’s disease (*A* and *B*), multiple sclerosis (*C* and *D*), and traumatic brain injury (*E* and *F*). For PD, control versus baseline-PD (C-P), control versus PD-2YFU (C-Pf), and baseline-PD versus PD-2YFU (P-Pf) discrimination tasks are shown separately. Classifiers were trained on brainstem bundles (n = 16) (black), *TractSeg* bundles known disease-related pathology in imaging literature for PD and MS or highest degree of overlap hemorrhagic lesions for TBI (n = 15 to 16) (green), hemispheric gray matter masks (n = 2) (purple), and a whole-brainstem mask (n = 1) (blue). AUC: Area under the receiver-operating characteristic curve, *n*: number of classifier features. GM: Gray matter, MLc: Medial lemniscus (caudal), SCP: Superior cerebellar peduncle, LFB: Lateral forebrain bundle, MHB: Mesencephalic homeostatic bundle, Bic: Brachium of the inferior colliculus, MLr: Medial lemniscus (rostral), MLF: Medial longitudinal fasciculus, CTG: Central tegmental tract PD: Parkinson’s disease, MS: Multiple sclerosis, TBI: Traumatic brain injury, PD-2YFU: Parkinson‘s disease two-year follow-up.

To assess BSBT’s clinical translatability, we evaluated voxel-wise average FA and volume alterations for each bundle ROI in AD, PD, MS, and acute severe TBI cohorts. FA is sensitive to a broad array of pathological changes to WM microstructure, and volume is commonly used to assess WM atrophy/damage (10, 43–48). FA and volume distributions for AD cohorts are shown in *SI Appendix*, Fig. S8, and for PD, MS, and TBI cohorts in Fig. 3. We evaluated BSBT’s discriminatory power by constructing linear discriminant analysis classifiers (LDA), henceforth referred to as “classifiers”, on the FA and volume of each brainstem bundle (separated by left–right subdivision, n = 16). We trained each classifier with leave-one-out cross-validation to discriminate healthy from pathological scans in each cohort. We compared areas-under-the-curve (AUC) from the receiver-operating characteristic (ROC) curves of each classifier to companion classifiers of other commonly segmented brain regions. These include hemispheric gray matter masks (n = 2), a whole-brainstem mask (n = 1) segmented using *SynthSeg* (49), and supratentorial WM bundles segmented with *TractSeg* (32) (n = 15 to 16). For more rigorous benchmarking, we chose subsets of *TractSeg* bundles with diffusion/volumetric changes associated with AD (50, 51), PD progression (10, 46, 52–57), and MS pathology (12, 58–61). For TBI, we chose *TractSeg* bundles with the greatest hemorrhagic lesion burden in our dataset. ROC curves for these classifiers in AD cohorts are shown in *SI Appendix*, Fig. S8, and in PD, MS, and TBI cohorts in Fig. 4. In the following sections, we report findings for each neurological disorder. We also provide ROC-AUC estimates for each individual brain region segmented for clinical analysis (all *TractSeg*, *SynthSeg* gray matter, and BSBT segmentations), in *SI Appendix*, Table S3. Details on MRI acquisition schemes, inclusion/exclusion criteria, subject information, and statistical analysis are in the *SI Appendix*, *Supplementary Text*.

#### Alzheimer’s disease.

Loss of WM integrity is a hallmark of AD, and while the majority of WM bundles affected are supratentorial, some WM changes have been reported in brainstem regions (11, 62, 63). We assessed brainstem bundle FA and volume alterations in 106 subjects diagnosed with AD or mild cognitive impairment (MCI) and 122 cognitively normal (control) subjects from the ADNI3 dataset. Notably, all brainstem bundles except for the left LFB and left MLr showed reduced volumes in AD/MCI, and the MHB displayed significant volume reductions (left *P* = 0.027, right *P* = 0.024). Individual bundle and ROC analyses are in *SI Appendix*, Fig. S8 and Table S3. FA and volume measurements for *TractSeg* bundles, hemispheric gray matter, and the brainstem mask are shown in *SI Appendix*, Figs. S9–S11.

#### Parkinson’s disease.

Prior studies, including dMRI analyses, have shown PD-related brainstem WM degeneration. This degeneration occurs in early-stage PD (9, 10, 46, 56, 64), progressing at variable rates. We assessed brainstem bundle FA/volume changes in 72 PD patients and 52 control subjects from the Parkinson’s Progression Markers Initiative (PPMI) dataset (65). For the PD group, we analyzed baseline (at time-of-diagnosis) and two-year follow-up (2YFU) scan changes. FA and volume measurements for *TractSeg* bundles, hemispheric gray matter, and the brainstem mask are provided in *SI Appendix*, Figs. S12–S14. The most significant FA reduction was in the LFB (left *P* < 0.001, right *P* = 0.005), Bic (left *P* < 0.001, right *P* = 0.01), and SCP (left *P* = 0.006, right *P* = 0.003) (Fig. 3*A*). Of those bundles, FA reduction in control-2YFU comparisons was significant in the left Bic (*P* = 0.006) and left LFB (*P* = 0.046). Volumetric analysis revealed significant volume loss in the MLr (left *P* = 0.002, right *P* = 0.021) between baseline-PD and 2YFU groups (Fig. 3*B*). ROC analysis showed that BSBT classification either outperformed or was comparable to the best classifier in control-2YFU FA discrimination (AUC = 0.69) and volume (AUC = 0.63). This performance was statistically significant with respect to *TractSeg* FA classification (AUC = 0.53, *P* = 0.024) (Fig. 4 *A* and *B*). Finally, ROC-AUC inspection for individual brain regions showed that some brainstem bundles such as the Bic, with AUC = 0.73 for baseline-PD-2YFU volume discrimination and AUC = 0.69 for control-2YFU FA discrimination, displayed similar power to basal ganglia structures, with the notable exception of the ventral diencephalon, which showed superior discriminatory power of all assessed brain structures (AUC = 0.96 for control-baseline-PD FA discrimination and AUC = 0.90 for control-2YFU volume discrimination) (*SI Appendix*, Table S3).

#### Multiple sclerosis.

WM demyelination and axonal breakdown are central pathological features in MS, with both supratentorial and infratentorial WM bundles exhibiting abnormalities in microstructure and morphology (12, 66). Given the clinical importance of brainstem involvement in MS, we assessed brainstem bundle FA and volumetric alterations in 98 MS patients and 50 healthy control subjects (67). Several brainstem bundles demonstrated significantly reduced FA in MS, including the SCP (left *P* < 0.001, right *P* < 0.001), MLc (left *P* = 0.044, right *P* = 0.020), left MLr (*P* = 0.025), and right MLF (*P* = 0.042) (Fig. 3*C*). Volume reductions were most pronounced in the MLc (left *P* = 0.003, right *P* = 0.011), LFB (left *P* = 0.003, right *P* = 0.013), and left Bic (*P* = 0.003) (Fig. 3*D*). FA/volume measurements for *TractSeg* bundles, hemispheric gray matter masks, and brainstem mask are shown in *SI Appendix*, Figs. S15-S17. The strongest discriminatory power between controls and MS was with BSBT FA classification (AUC = 0.80), statistically outperforming hemispheric gray matter classification (AUC = 0.67, *P* = 0.027) (Fig. 4*C*). Volume classification resulted in similar performances between BSBT and whole-brainstem classifiers (both AUCs = 0.65), with *TractSeg* classification displaying the greatest discriminatory power (AUC = 0.73) (Fig. 4*D*). Individual-region ROC analysis indicated that the BSBT-segmented SCP showed similar or superior discriminatory power in FA classification (AUC = 0.78) to supratentorial structures most-known to be affected by MS, including the corpus callosum (AUC = 0.71 for FA), optic radiation (AUC = 0.79 for FA), and thalamo-occipital radiations (AUC = 0.78 for FA) (*SI Appendix*, Table S3).

#### Traumatic brain injury.

Severe TBI can lead to multifocal WM disconnection and result in disorders of consciousness, such as coma and vegetative state (68–70). We analyzed FA and volume changes in 17 patients with acute severe TBI and 29 control subjects to detect early brainstem bundle integrity disruption. MRI data from these subjects were used in prior studies of brainstem alterations in TBI (22, 39, 71–73). Clinical/demographic information for TBI patients are in *SI Appendix*, Table S4. BSBT analysis revealed no significant volume changes between TBI and control cohorts, while the TBI group showed reduced FA for the majority of brainstem bundles (Fig. 3 *E* and *F*). FA was significantly lower in three brainstem bundles: bilateral LFBs (left *P* = 0.001, right *P* = 0.010), bilateral MLFs (left *P* = 0.033, right *P* = 0.009), and left MLc (*P* = 0.012). Brainstem bundles showed variable overlap with hemorrhagic lesions traced in corresponding susceptibility-weighted images (SWI) in TBI patients, with the CTG displaying the highest lesion overlap score (Dice = 0.113) (*SI Appendix*, Fig. S18). SWI lesion localization and tract-overlap analysis are described in the *SI Appendix*, *Supplementary Text* and illustrated in *SI Appendix*, Fig. S19. SWI scans were also used to select *TractSeg* bundles for ROC analysis; 16 *TractSeg* bundles with the greatest lesion overlap Dice score were used for benchmarking.

The BSBT FA classifier outperformed all other classifiers (AUC = 0.77), with statistical significance against the hemispheric gray matter FA classifier (*P* = 0.035) (Fig. 4 *E* and *F*). ROC-AUC assessment for all individual brain regions revealed that the LFB displayed the greatest discriminatory power (AUC = 0.82 for FA) among all brainstem bundles. However, supratentorial tracts such as the corpus callosum (AUC = 0.86 for FA), fronto-pontine tract (AUC = 0.94 for FA), striato-fronto-orbital tract (AUC = 0.96 for FA), striato-prefrontal tract (AUC = 0.96 for FA) and thalamo-prefrontal tract (AUC = 0.95 for FA) displayed the greatest individual discriminatory performance among all segmentations (*SI Appendix*, Table S3). FA/volume measurements for *TractSeg* bundles, hemispheric gray matter, and brainstem mask are provided in *SI Appendix*, Figs. S20-S22.

### Longitudinal Brainstem WM Analysis in Traumatic Coma Recovery.

**Fig. 5.**
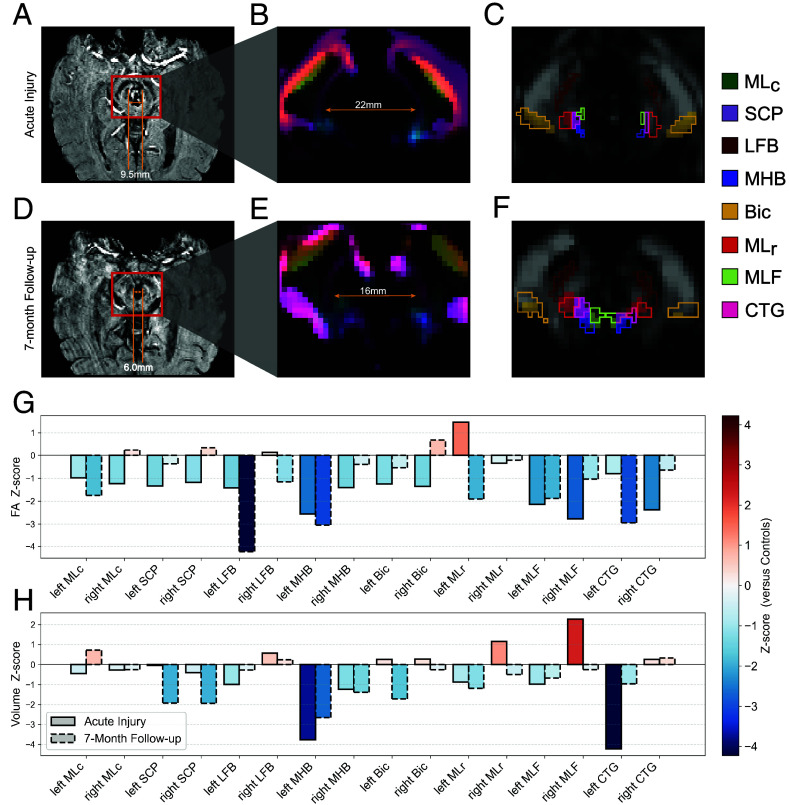
Longitudinal brainstem WM alterations in a patient with severe traumatic brain injury. (*A*) SWI scan during the acute injury phase of a patient who suffered a traumatic coma from a severe TBI. (*B*) PFM channel from the corresponding dMRI, showing significant mass-effect causing displacement, but not direct lesioning, of brainstem bundles due to the development of an acute traumatic midbrain hemorrhage. (*C*) The FA channel with WM bundle segmentations from a CNN using the PFM (outlined), overlaid with semitransparent segmentations from a CNN without the PFM. The patient had functional recovery and underwent follow-up scanning at 7 mo, which showed a significant decrease in midbrain lesion volume (*D*). The corresponding PFM channel (*E*) showed a counterdisplacement of brainstem bundles proximal to the lesion toward the midline, and BSBT segmentations from a CNN both with and without the PFM revealed coherent bundle reconstructions (*F*). Also shown are FA (*G*) and volume changes (*H*) as compared to corresponding per-bundle means and SD of FA/volume from the TBI dataset control subjects. GM: Gray matter. MLc: Medial lemniscus (caudal), SCP: Superior cerebellar peduncle, LFB: Lateral forebrain bundle, MHB: Mesencephalic homeostatic bundle, Bic: Brachium of the inferior colliculus. MLr: Medial lemniscus (rostral), MLF: Medial longitudinal fasciculus, CTG: Central tegmental tract.

We performed longitudinal BSBT analysis in a 29-y-old man with an acute disorder of consciousness caused by severe TBI (P15 from the TBI dataset, see *SI Appendix*, Table S4). The patient’s coma was attributed to a large midbrain hemorrhagic lesion. Specifically, MRI scanning on day 7 post-TBI revealed an acute traumatic hemorrhage along the entire midsagittal extent of the midbrain (Fig. 5*A*), which typically results in a poor long-term outcome (74). We chose this patient for morphometry for two reasons: first, he had the largest brainstem lesion of any patient in our acute severe TBI study (ClincialTrials.gov NCT03504709), and second, he regained consciousness, communication, and partial functional independence by 7 mo postinjury (Glasgow Outcome Scale-Extended score = 5) (71).

To identify the impact of the midbrain lesion on WM mapping, we segmented the patient’s brainstem bundles using the BSBT CNN with and without a PFM channel. Visual inspection of PFM intensities showed that WM bundles near the lesion periphery were laterally displaced but not disconnected by the lesion (Fig. 5*B*). Segmentation without the PFM (only with low-b and FA inputs) resulted in near-absent bundle labels on the radiologic left side of the midbrain, indicating severe tract disconnection (Fig. 5*C*). In contrast, segmentation with the PFM channel detected these bundles, revealing their preservation and potentially explaining the patient’s unexpected long-term functional recovery. A 7-mo follow-up scan revealed a decrease in lesion volume from 1,962 mm^3^ (acute) to 679 mm^3^ (follow-up) (Fig. 5*D*), and the PFM showed that displaced bundles shifted back toward their expected neuroanatomic locations (Fig. 5*E*). Segmentation with and without the PFM showed bilateral WM bundle reconstructions at 7-mo follow-up (Fig. 5*F*). Collectively, these longitudinal observations imply that PFM information aids in WM bundle reconstructions in heavily lesioned and deformed brainstem regions.

Quantitative tract-wise analysis confirmed that several WM bundles displayed marked FA/volume decreases relative to controls in the same dataset (Fig. 5 *G* and *H*). The left MHB, right CTG and MLF showed the most severe FA and/or volumetric reductions in the acute phase (Z-scores < −2), consistent with their relative proximity to the lesion. At the 7-mo follow-up, FA values in several bundles partially increased, with corresponding volume increases, though FA and/or volume remained persistently reduced in some tracts (e.g., the left MHB, left CTG and left LFB).

## Discussion

We developed BSBT, an unsupervised algorithm that automatically segments brainstem bundles in dMRI of the human brain. Segmentations showed cross-dataset accuracy and reliability through comparison with gold-standard ex vivo manual annotations, and silver-standard annotations in single- and multishell in vivo dMRI. We demonstrate BSBT’s clinical translatability and provide proof-of-principle evidence for its utility as a complementary method that contributes tract-resolved WM information and aids in assessing neurological conditions differentially affecting the brainstem. We release BSBT as a tool to map human brainstem WM in healthy and diseased brains (github.com/markolchanyi/BSBT).

BSBT addresses a key gap in brainstem imaging because it is capable of automatically segmenting small brainstem bundles without manual intervention across dMRI domains. BSBT showed high segmentation accuracy at varying resolutions (0.7 to 2 mm), with Dice ranging from 0.62 to 0.70 and subject-averaged HD not exceeding 2.5 mm. These metrics are comparable to state-of-the art segmentation algorithms of similarly sized brain regions such as hypothalamic, thalamic, and brainstem nuclei (39, 75–77), which were segmented at similar-or-finer spatial resolutions.

Ablation and test–retest analyses demonstrated BSBT’s reliability and consistency. Accuracy reduction occurred in at least one validation dataset following the removal of each core BSBT component, both within the CNN (CRF/attention gating mechanism) and for input features (PFM removal/replacement with V1). We also varied the CNN input and training loss (*SI Appendix*, Fig. S7). Similar validation accuracy at convergence across diffusion scalar inputs, and lower accuracy with boundary-based training loss, further suggests that BSBT’s gains are driven primarily by PFM contrast and architecture modifications to localize small/clustered structures, rather than choosing optimal diffusion scalars or loss function variants (*SI Appendix*, Fig. S1). On this basis, and for parity with prior work (76, 77), we retained our CNN input configuration and training regime. However, we acknowledge that more robust multishell metrics like NODDI/kurtosis (78, 79) or fiber orientation distribution-based channels, which can also provide partial-voluming estimates for degraded dMRI, remain attractive variants for future work. Another important future direction is incorporation of statistical or deep-learning-based harmonization to further mitigate interdomain variability in heterogenous dMRI datasets (80). We deliberately avoided harmonization to demonstrate native generalizability across both single- and multishell dMRI of variable angular resolution.

Although segmentation reliability for small regions is expected to be low (due to fluctuations in measured volume from noise), test–retest analyses yielded high intrasubject ICC (ICC > 0.8) across all bundles, except for the ML_C_ and left MHB (ICC > 0.7). This implies that BSBT uses anatomically plausible features and not structures arising from noise. Negligible ICC-volume correlation further suggests that each segmented structure is above signal-to-noise-ratio limits in HCP-quality data.

BSBT’s utility as a research and clinical add-on tool is highlighted by single-tract analysis and multitract classification across AD, PD, MS, and TBI. Specifically, we explore how neurodegenerative diseases and acute brain injury exhibit differential effects on brainstem WM integrity and assess the discriminatory power of brainstem bundles in classification tasks for each disorder. We provide discriminatory power estimates for all individual segmented brain regions, which confirm strong, disease-specific supratentorial patterns of FA and/or volumetric changes. These include but are not limited to AD-related hippocampal atrophy, and FA reduction in the basal ganglia/diencephalon in PD, optic radiation in MS and corpus callosum in TBI. Nonetheless, subsets of BSBT-segmented bundles showed comparable discriminatory performance across PD, MS, and TBI. This further prompts us to position BSBT not as an isolated clinical tool, but as a key adjunct that aids current diagnostic imaging methods by providing fine-grained assessment of brainstem WM structure and, in some cases, longitudinal information.

Due to the relative lack of AD-related brainstem pathology (11, 63, 81–84), especially in MCI stages, we aimed for AD analysis to provide a calibration for effect sizes in cohorts with more prominent brainstem involvement. We therefore included ADNI3 analysis as a low-expected-effect comparator, especially when supratentorial measures are known to exhibit stronger classification performance (85). This analysis only revealed MHB volume reduction. Interestingly, the MHB connects several brainstem arousal/homeostatic nuclei to regions with AD-related degeneration patterns, including the basal forebrain, hippocampus, and entorhinal cortex (15, 86–88). However, especially at earlier disease stages, this finding is best corroborated by concurrent microstructural (i.e., FA) changes. We did not identify FA changes in any brainstem bundles, whereas supratentorial bundles displayed more widespread FA/volume reduction (*SI Appendix*, Fig. S9), providing the most powerful LDA classification. These findings strengthen confidence that BSBT does not generate spurious effects in disease cohorts with limited brainstem involvement. Accordingly, we view BSBT in this AD analysis, and in the clinical assessments as-a-whole, as complementary to established clinical biomarkers, with its primary value in enabling mechanistic, morphological, and longitudinal studies of distinct brainstem WM pathways rather than as a standalone diagnostic and/or prognostic method.

In contrast to AD, PD is characterized by α-synuclein accumulation in the brainstem and loss of dopaminergic neurons in the substantia nigra among other regions. While brainstem structural degeneration is documented in prior literature (89, 90), affected WM pathways have not been comprehensively classified. One study reported dMRI-based PD subtyping in multiple small brainstem bundles extracted from atlas coregistration (91). In our analysis, the most pronounced finding was bilateral LFB, Bic and SCP FA reduction between baseline-PD and 2YFU scans. Bilateral changes in these bundles were individually observed in many PD subjects, indicating that FA reduction is not driven by a small subset of subjects, but rather cohort-wide degeneration (*SI Appendix*, Fig. S23). These findings are consistent with brainstem involvement in the degeneration of nigral, basal ganglia, forebrain, cerebellar, and cortical targets associated with these bundles (17, 89, 92–95). FA patterns followed a biphasic trajectory, increasing between controls and baseline-PD, then decreasing in 2YFU. This pattern has been previously described (90) and may explain the pronounced FA reduction between PD groups relative to controls. The left LFB/Bic also showed significant FA reduction between controls and 2YFU groups, with the corresponding classifier displaying the highest discriminatory power (AUC = 0.69). Collectively, these findings highlight how brainstem bundle diffusion metrics may serve as early-stage PD biomarkers, providing both detection and disease progression information.

MS often presents as multifocal demyelination and axonal degeneration with frequent brainstem WM involvement, particularly within oculomotor/somatosensory pathways (12, 61, 96). Tract-resolved, group-wise brainstem WM analysis remains uncommon. Although MS lesions are prevalent in the brainstem, prior imaging studies on individual WM pathways are limited to large bundles such as corticospinal tracts and cerebral peduncles (59, 61, 66). With BSBT, we show robust FA reductions in the SCP, both ML subdivisions and MLF. These findings align with prior lesion studies and well-established MS clinical phenotypes: SCP lesions with cerebellar ataxia (97, 98), ML lesions with sensory impairment (99, 100), and MLF lesions underpinning internuclear ophthalmoplegia (101). Volumetric loss was most evident in the MLc, LFB, and Bic, suggesting downstream degeneration in posterior tegmental and cerebello-thalamo-cortical pathways, as previously reported (98, 102). Stronger FA effects in the SCP, ML, and MLF versus more pronounced volumetric atrophy in the LFB and Bic likely reflects varying temporal windows of MS progression (i.e., acute demyelination versus later-stage axonal loss and gliosis). BSBT FA classification provided the greatest discriminatory performance (AUC = 0.80), suggesting that disease-relevant tissue changes are concentrated within discrete WM pathways rather than being evenly distributed across the brainstem. In contrast, performance among volume-based classifiers was comparable, consistent with volumetry capturing a more global, cumulative burden that is less pathway-specific. Taken together, these results indicate that BSBT-derived diffusion metrics capture MS-specific microstructural injury with comparable fidelity to whole-region infratentorial averages (i.e., whole-brainstem) and even supratentorial bundles. This motivates the use of bundle-specific brainstem metrics as a complementary marker to supratentorial measures in diagnosis, prognosis, and disease monitoring for MS.

The application of BSBT to patients with acute severe TBI demonstrates two additional features of the algorithm: its ability to detect focal brainstem bundle alterations and to identify bundles in the presence of deformation/lesioning. Pathological and neuroimaging studies suggest that axonal injury in acute severe TBI leads to FA reduction with regional variability, and commonly observed diffusivity changes near brainstem arousal centers (68, 103–107). Our prior analyses of brainstem arousal nuclei in close neuroanatomic proximity to the segmented WM bundles have also shown MRI-based changes associated with lesion burden and behavioral metrics in the same TBI cohort (39, 73). Accordingly, we found statistically significant FA reduction in the LFB, MLF, and left MLc. This likely contributed to the high predictive power of BSBT FA classification (AUC = 0.77), which outperformed every competing classifier. While there is strong evidence that injury to brainstem arousal nuclei can cause coma (14, 22, 73), little is known about the clinical correlates of associated WM. The LFB contains connections between arousal nuclei and parietotemporal default mode network regions and is believed to be a key pathway for arousal-awareness integration in human consciousness (17). BSBT identification of LFB FA reduction thus warrants future investigation into the role of individual brainstem WM pathways in coma pathogenesis.

We demonstrated that BSBT can identify preserved brainstem bundles in a severe TBI patient with a large brainstem lesion who experienced full functional recovery. In the acute scan, we identified brainstem bundles with high deformation by mass effect, highlighting the PFM’s crucial role. Quantitatively, most brainstem bundles displayed marked FA/volumetric reductions relative to controls, consistent with acute injury. At the 7-mo follow-up scan, when the lesion size had decreased, BSBT segmentation, both with and without PFM channels, reconstructed bundles on both left and right lesion margins. PFM-based segmentations in the acute scan were therefore anatomically founded and not false-positive reconstructions. Furthermore, FA and/or volume partially normalized in several bundles proximal to the lesion—including bundles that contribute to modulating consciousness—the MHB and CTG. Taken together, these observations support two inferences with prognostic value. First, baseline structural preservation of bundles with lateral displacement serves as an early indicator of functional recovery. Second, longitudinal realignment with FA/volume improvement provides evidence of bundle reorganization that may support clinical recovery. We therefore postulate that the high sensitivity of the PFM, coupled with the proposed CNN segmentation model, has substantial prognostic potential by identifying preserved brainstem bundles that can facilitate coma recovery.

The BSBT evaluation and its applications to patients with neurological disorders is limited by model design constraints and the overall clinical dataset composition. Our model-specific limitation mainly pertained to CNN training, which was limited to 30 subjects from a single dataset annotated by a single rater. To combat overfitting, we aggressively augmented our training data but still observed noticeable accuracy differences between validation datasets. While this is indicative of model parameters overly tuned to training dataset-specific features, test–retest ICCs remained high and segmentation performance was consistent across a large resolution span in our resampling task (*SI Appendix*, Fig. S6). Nonetheless, we observed systematically lower Dice in ex vivo test data, likely due to high domain shift. *Postmortem* fixation restricts free-water diffusion in brain tissue, drastically altering diffusion signal profiles (108) and complicating modeling ex vivo contrast from in vivo training data. Incorporating other datasets for training, such as ex vivo, low-field, and low angular resolution dMRI, may increase segmentation robustness.

BSBT ultimately inherits the quality of its input scans and does not replace artifact correction. dMRI with severe motion, pulsatile ghosting, and/or uncorrected susceptibility may still fail. Furthermore, partial-voluming remains a constraint at clinical resolutions, where attention gating, CRF sharpening, and data augmentation mitigate but do not eliminate these effects. Due to the relatively small size of brainstem bundles, even single-voxel drifts of segmentations due to scanner noise, distortions, and/or spatial resolution can potentially miss the true bundle location, making individual-level interpretations especially variable. We therefore recommend stringent quality control prior to segmentation and caution against its use in heavily degraded dMRI.

The primary limitations for all clinical datasets we analyzed were class imbalance, high feature numbers relative to sample sizes (posing a risk of classification bias), and overall scan quality. We used nonparametric statistical models with false-discovery rate correction for more rigorous statistical analysis, and implemented a simple linear model (i.e., LDA) for classification to avoid overfitting. However, larger sample sizes are necessary to validate our clinical findings, especially in the setting of multibundle analysis. At this clinical in vivo scanning quality and resolution span, we have found that overall noise levels more-often produce failed segmentations of one or more brainstem bundles, and gray-white partial-voluming inevitably leads to inclusion of proximal gray matter tissue within each bundle ROI and confounds identification of pure-WM pathology. This is particularly pertinent in the presence of gray matter alterations adjacent to the segmented bundles, such as PD-related α-synuclein inclusions in the brainstem tegmentum and substantia nigra (which borders the LFB) (9, 109), or direct injury to brainstem arousal nuclei in traumatic coma (22, 68, 73), which are adjacent to most BSBT-segmented bundles. While the current version of BSBT utilizes dMRI-exclusive WM contrast, future validation studies should evaluate whether adding brainstem gray matter context (such as probability maps from arousal nuclei (39)) can refine tract specificity near gray-WM interfaces to improve segmentation, especially in the setting of partial-voluming.

Finally, we note several dataset-specific limitations. For PD subjects, low signal-to-noise ratios likely influenced both excluded subject numbers and statistical analysis, as outlined in the *SI Appendix*, *Supplementary Materials* and Fig. S24. We applied Gaussian smoothing to more noise-susceptible FA maps (110) to attempt to match signal-to-noise ratios of the other clinical datasets. Nonetheless, spurious statistical changes may have arisen due to noisy FA measurements, highlighting the need for further evaluation with alternative acquisitions and denoising methods. AD, MS, and TBI analyses were likely hindered by the variable distribution of disease stages. To increase sample size, we grouped AD and MCI subjects to an “AD/MCI” supergroup at the potential expense of capturing longitudinal variability in FA and volume measurements (63, 111). MS control and patient groups had the largest age gap (mean age: controls = 57.1 y, patients = 47.2 y) and spread (age SD: controls = 16.7, patients = 11.2), introducing the possibility that some group differences reflect age-related variation rather than disease progression. Furthermore, MS patients were not scanned at specific disease stages/degrees of lesion burden [see inclusion criteria from Fiscone et. al. (67)], which likely contributed to the heterogeneity of FA-based and volume measurements. Similarly, for TBI subjects, the principal limitation was variable injury-to-imaging intervals. Because dynamic microstructural alterations occur within hours-to-days of a TBI, time of imaging becomes a critical factor for diffusion measurements (112). In future work, standardizing injury-to-imaging intervals can provide deeper insights into the temporal evolution of brainstem dysfunction and recovery in severe TBI.

In summary, we present BSBT, a brainstem bundle segmentation algorithm that we rigorously validated with high-resolution ex vivo and in vivo dMRI, and analyzed in multiple neurological disorders. This automated approach to studying the brainstem stands to streamline neuroimaging research and promote clinical investigation of brainstem WM morphology, integrity, and connectivity in healthy individuals and in those with neurologic disease. While the method currently segments eight brainstem bundles, there remain many avenues for extending segmentation to additional bundles, which will further advance brainstem connectivity mapping.

## Materials and Methods

### Probabilistic Fiber Map Construction.

**Fig. 6.**
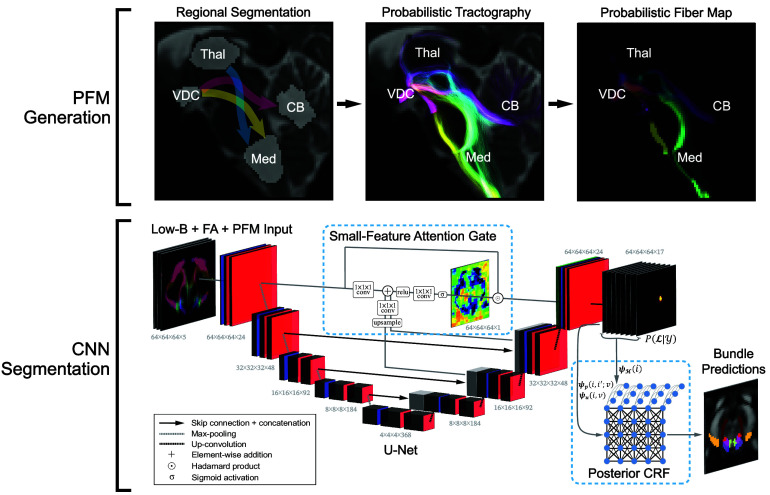
Probabilistic fiber map construction and neural network segmentation framework. (*Top*) Probabilistic streamlines are seeded between four ROIs that lie adjacent to the rostral brainstem: The Ventral Diencephalon (VDC), Thalamus (Thal), Cerebellar gray matter (CB), and Medulla Oblongata (Med). VDC, Thal, and CB masks in this figure are dilated by a 3-voxel kernel for clearer visualization. Streamlines are histogram-normalized and combined into a single 3-channel PFM. (*Bottom*) The PFM is combined with low-b and FA channels as part of a 5-feature input to a U-Net CNN, which is modified with an attention gating mechanism placed on the three highest-resolution encoding/decoding layers. The CNN *SoftMax* output ($P\left(L|Y\right)$) is processed by the CRF with unary ($\psi_{u}$), pairwise ($\psi_{p}$), and label entropy ($\psi_{p}$), potentials to output refined brainstem bundle segmentations. ROI: Region of interest, CNN: Convolutional neural network, CRF: Conditional Random Field, PFM: Probabilistic Fiber Map, FA: Fractional Anisotropy.

The workflow to generate the PFM is illustrated in Fig. 6. We enable users to only input a dMRI volume without requiring companion T1/T2 sequences, which is common in dMRI segmentation algorithms (30, 76), by performing all processing in dMRI space. The three PFM channels are composed of probabilistic streamlines seeded between the thalamus and medulla (channel 1), between the cerebellar gray matter and ventral diencephalon (channel 2), and between the ventral diencephalon and medulla (channel 3). PFM intensities were directly correlated with MRI and histological contrast to determine ground-truth brainstem bundle locations in our ex vivo cases, five of which were used in our prior study for probabilistic atlas construction of gray matter ascending arousal network nuclei (*SI Appendix*, Figs. S1, and S4–S7; see *SI Appendix*, Table S1) (39). All dMRI preprocessing steps and further details on PFM construction are described in the *SI Appendix*, *Supplementary Text*.

#### Convolutional neural network architecture.

We used a U-Net CNN architecture previously adapted for dMRI segmentation tasks (77, 113). The CNN comprises five resolution levels, each containing $24\times 2^{n-1}$ features (n: level number), and two convolutional layers with 3 × 3 × 3 kernels and Exponential Linear Unit activation functions. The resolution is halved at every level with max-pooling. The CNN takes in a five-channel input (low-b, FA, and PFM). Input channels are resampled to 1 mm isotropic resolutions and precropped around the pontine ROI center-of-mass (segmented by the brainstem subfield segmentation algorithm (34)). A schematic overview of the full CNN model is illustrated in Fig. 6. Further details on model training and inference, including train-time data augmentation, is in the *SI Appendix*, *Supplementary Text*.

We use an attention-gating mechanism, which is commonly used in biomedical image segmentation (114–117), to amplify small features. We integrate inputs from multiple decoder layers in our attention gate to capture feature information across spatial scales. Let the CNN encoder/decoder layers be defined as $F_{e}^{n}$/$F_{d}^{n}$ where n∈1...N is the CNN layer number (ordered from fine/high-resolution to coarse/low-resolution), where $F_{e}^{n},F_{d}^{n}\in R^{N_{d}\times N_{d}\times N_{d}\times N_{f}}$ s.t. $N_{d}=64*2^{1-n}$ and $N_{f}=24*2^{n-1}$. We only implement gating for the $F_{e}^{1}$ − $F_{d}^{1}$ skip connection. We integrate gating signals from all decoder layers except for the coarsest layer (due to its overly “blocky” four voxel feature representation in each spatial dimension). Finally, rather than downsampling the input encoder layer ($F_{e}^{1})$ to the resolution of decoder gating signals, we upsample all gating signals to the resolution of $F_{e}^{1}$ (and by extension $F_{d}^{1}$) to preserve fine-scale features. These implementations aim to create a more interpretable gating model by additively combining multiple decoder gating signals. The gating signals are transformed with a hyperbolic tangent activation function prior to being added to the incoming layer for weight normalization, which allows for both positive and negative outputs to mimic receptive field activation/suppression. We define the attention gating mechanism $\Phi_{\mathrm{att}}(\cdot )$ as:

$$\begin{array}{c} \Phi_{\mathrm{att}}\left(F_{d}^{1};\;F_{e}^{1},F_{d}^{n}\right)=F_{d}^{1}\circ \sigma \left\{\varphi_{a}⊗relu\left(F_{e}^{1}+\sum_{n=2}^{N}\varphi_{n}⊗tanh\left(U\left[F_{d}^{n}\right]\right)\right)\right\}, \end{array}$$

where ∘ is the Hadamard product, ⊗ is the three-dimensional convolutional operator, φ is a 1×1×1 convolutional kernel, $U\left(\cdot \right)$ is an upsampling operation for match the dimensionality $F_{e}^{1}$, relu(·) is a rectified linear unit, $tanh\left\{\cdot \right\}$ is the hyperbolic tangent function and $\sigma \left\{\cdot \right\}$ is a sigmoid activation function. The effects of each attention-gate modification are visualized with a representative ex vivo subject in *SI Appendix*, Fig. S25.

#### CRF design.

The background label probabilities from the *SoftMax* CNN layer can dominate and dilute the probabilities of brainstem bundle labels, even with the use of a Dice penalty during CNN training. This is more evident in domains differing from the training dataset, such as low-resolution in vivo and ex vivo dMRI. We employ a label probability enhancement strategy for the *SoftMax* CNN layer with a semidense CRF (118). CRF refinement is employed to increase label probabilities near bundle edges, which are often inpainted by background. Let the CNN input volume $Y\in R^{64\times 64\times 64\times 5}$ consisting of $N=64^{3}$ voxels in a uniform grid, a corresponding ground-truth label map $L\in R^{N\times V}$ consisting of V labels, such that, for a location (i.e., voxel) i, $L=\left\{l_{i}:i=1...N|l_{i}\in R^{V}\right\}$. We infer the refined label probabilities $P\left(L|Y\right)$ with the CNN, which are originally approximated with the *SoftMax* output $S\in R^{N\times V}$. The joint posterior probability distribution can be written as:

$$P\left(L|Y\right)=\prod_{i=1}^{N}\prod_{v=1}^{V}P\left(l_{i,v}|Y\right)\approx \prod_{i=1}^{N}\prod_{v=1}^{V}S\left(i;v\right).$$

For CRF refinement, we reformulate $P\left(L|Y\right)$ as a Gibbs distribution with the CRF energy functional $E\left(L;.\right)$, such that:

$$P\left(L|Y\right)\propto \exp\left[-E\left(L;Y\right)\right].$$

We model $E\left(L;.\right)$ as a linear combination of the negative log-likelihood $\psi_{u}$, log-pairwise potential $\psi_{p}$, and label entropy regularizer $\psi_{H}$ (118, 119). We refine $P\left(L|Y\right)$ to better capture label interactions through Maximum a Posteriori inference, which is equivalent to minimizing $E\left(L;.\right)$ to determine the updated labels $L_{CRF}$:

$$L_{\mathrm{CRF}}=\underset{L}{\text{argmin}}\;E\left(L;Y\right),$$

$\psi_{u}$ (the label probability negative log-likelihood) and $\psi_{H}$ are defined voxel-wise and separately for each label (where we assign v=1 as the background label) as:

$$\psi_{u}(i,v)=-\log\left[P\left(l_{i,v}|Y\right)\right],$$

$$\psi_{H}\left(i\right)=-E_{v,v\neq 1}\left[\log\left\{P\left(l_{i,v}|Y\right)\right\}\right],$$

$\psi_{H}$ regularizes certainty (i.e., distinctness) for foreground labels without background interference. This preserves structure by maintaining sharp boundaries between adjacent labels, while allowing label probabilities to dominate in regions of background inpainting (i.e., in regions of anatomically distinct foreground with dominant background posteriors). We decompose $\psi_{p}$ into spatial ($\psi_{p}^{sp}$) and intensity ($\psi_{p}^{in}$) components to sensitize the CRF to intensity fluctuations in adjacent voxels. We define $\psi_{p}$ for the voxel pair $\left(i,i^{\prime }\right)$, around a local neighborhood $X_{i}=\left\{i^{\prime }:{∥i-i^{\prime }∥}_{2}^{2}\leq x\right\}$ as:

$$\psi_{p}\left(i,i^{\prime };v\right)=\psi_{p}^{sp}\left(i,i^{\prime };v\right)\;*\;\psi_{p}^{in}\left(i,i^{\prime };v\right)$$

$$=RBF\left(i,i^{\prime };{v,\sigma }_{b}\right)\;*\;RBF\left(P\left(l_{i}|Y\right),P\left(l_{i^{\prime }}|Y\right);v,\sigma_{b}\right)$$

$$\begin{array}{c} =\exp\left[-\frac{{∥i-i^{\prime }∥}_{2}^{2}+{\left\{P\left(l_{i,v}|Y\right)-P\left(l_{i^{\prime },v}|Y\right)\right\}}^{2}}{2{\sigma_{b}}^{2}}\right], \end{array}$$

where ${∥\cdot ∥}_{2}$ is the Euclidean norm, and $\mathrm{RBF}\left(\cdot ;\sigma \right)$ is a Radial Basis Function operator parameterized by σ. We assume that $\psi_{p}^{sp}$ and $\psi_{p}^{in}$ possess equal importance such that $\mathrm{RBF}\left(\cdot ;\sigma_{b}\right)$ is parameterized by a common $\sigma_{b}$. To exploit the additive nature of potentials in log-space, we express the total CRF energy penalty as:

$$E_{\mathrm{comp}}\left(L;S,\sigma_{b},\sigma_{K}\right)=w_{u}\psi_{u}+\frac{1}{2\pi }\log\left[\psi_{p}\right]+\lambda \psi_{H},$$

where we collapse the pairwise indicator function and approximate $\psi_{p}^{sp}$ by convolving S with an RBF kernel $K_{\sigma_{K}}$ parameterized by $\sigma_{K}$ which encapsulates the CRF neighborhood size. This convolution decreases GPU-accelerated run-time by reducing the log-pairwise potential to:

$$\begin{array}{c} \log\left[\psi_{p}\left(i,i^{\prime };v\right)\right]\approx -\frac{{\left\{S_{\sigma_{K}}\left(i^{\prime };v\right)-S_{\sigma_{K}}\left(i;v\right)\right\}}^{2}}{2\sigma_{b}\sigma_{K}}, \end{array}$$

where:

$$S_{\sigma_{b}}\left(\cdot ;v\right)=K_{\sigma_{K}}⊗S\left(\cdot ,v\right).$$

The explicit form of the CRF energy penalty used for computation ($E_{\mathrm{comp}}$) and applied to S can therefore be expressed as:

$$\begin{array}{c} E_{\mathrm{comp}}\left(L;S,\sigma_{b},\sigma_{K}\right)\approx -\sum_{i=1}^{N}\sum_{v=1}^{V}\left\{\sum_{\{i,i^{\prime }\}\in X_{i}}\frac{{\left\{S_{\sigma_{K}}\left(i^{\prime };v\right)-S_{\sigma_{K}}\left(i;v\right)\right\}}^{2}}{4\pi \sigma_{b}\sigma_{K}}+\log\left[S\left(i,v\right)\right]\left(1_{\left\{v\neq 0\right\}}*\lambda S\left(i,v\right)+w_{u}\right)\right\}, \end{array}$$

where $1_{\left\{\cdot \right\}}$ is the indicator function. We set the $\sigma_{K}$ to five voxels, and neighborhood span of $X_{j}$ to three voxels to permit faster computation. The CRF energy penalty above is solved with iterative mean-field approximation over a small number of iterations with a fixed run-time to avoid overfitting.

## Supplementary Material

Appendix 01 (PDF)

Dataset S01 (XLSX)

## Data Availability

All code used for probabilistic mapping, segmentation and statistical analysis. Data have been deposited in Github (https://github.com/markolchanyi/BSBT). Ex vivo diffusion and FLASH sequences, and representative PFM reconstructions. All digitized histopathological sections.] data have been deposited in OpenNeuro, Biolucida (https://doi.org/10.18112/openneuro.ds006001.v1.0.2, https://histopath.nmr.mgh.harvard.edu/images/?page=images&selectionType=collection &selectionId=50). Some study data available (All TBI-related diffusion and/or SWI data will be shared by request in an anonymized format.) (17).
